# Science Cafés: Transforming citizens to scientific citizens—What influences participants’ perceived change in health and scientific literacy?

**DOI:** 10.1017/cts.2016.24

**Published:** 2017-04-19

**Authors:** Syed M. Ahmed, Mia DeFino, Emily Connors, Alexis Visotcky, Anne Kissack, Zeno Franco

**Affiliations:** 1 Department of Family and Community Medicine, Medical College of Wisconsin, Milwaukee, WI, USA; 2 Clinical and Translational Science Institute, Medical College of Wisconsin, Milwaukee, WI, USA; 3 Department of Biostatistics, Medical College of Wisconsin, Milwaukee, WI, USA; 4 Aurora Health Care, Aurora Research Institute, Milwaukee, WI, USA

**Keywords:** Health literacy, scientific literacy, scientific citizen, community engagement, dissemination

## Abstract

**Introduction:**

Science Cafés facilitated by the Clinical and Translational Science Institute of Southeast Wisconsin seek to increase health and scientific literacy through informal conversation between researchers and community members. The goal was to understand what factors have the greatest influence on attendees’ perceived changes in health and science literacy levels (PCHSL) to increase impact.

**Methods:**

Previous research established the evaluation used in the Science Cafés to measure PCHSL. In this study, comparisons were made between (1) 2 different approaches to Science Cafés (Genomics Science Cafés or Health Science Cafés) and (2) regression models to show which factors best predicted PCHSL.

**Results:**

The approach of the Genomics Science Cafés series to Science Cafés showed a larger impact on PCHSL. Regression models suggest SES and education significantly contributes to PCHSL.

**Conclusions:**

Insights for program development to have greater impact on PCHSL were identified. Continuing to optimize dissemination of research findings to the public is essential for improving community health and well-being.

## Introduction

Health science awareness is increasingly important to community health and primary care as individuals seek information related to their health from diverse sources [1–4]. Even with diverse sources available, the ability of individuals to understand, measure the credibility of the source/information, and use the information is still highly dependent on their formal education, health status, and health literacy [1, 5]. US subpopulations have different levels of access to health science information and differ in their ability to use that information to directly benefit their health outcomes. In a recent study by Rooks *et al*. [6], African Americans and Latinos were significantly more likely than Whites to use the information they found to make changes to maintain their health. As science communication and public dissemination of research findings takes deeper root, understanding how different groups process and use health science information is critical to community and personalized health information delivery [7].

Increasing health science awareness^1^ and providing resources to underserved populations is a goal of the Clinical and Translational Science Institute (CTSI) in Milwaukee, WI [8]. The CTSI is 1 of 62 awards made by the National Institutes of Health’s Clinical and Translational Science Award program, which seeks to transform biomedical research processes in part by engaging communities in research [9]. Science Cafés serve as opportunities for researchers to have a conversation with community designed to increase health and science literacy, by disseminating research results in an approachable format. In the context of community health and science communication, Science Cafés provide an informal learning environment for the public as well as an opportunity for researchers to hone key communication skills with the public [10, 11].

As education, health literacy, and health status all directly impact health disparities; it was of interest to determine how best to reach underserved populations and what factors had the greatest influence on their health and scientific literacy [2, 5]. Health-related disparities refer to differences between groups of people; these differences can affect how frequently a group gets sick, how often a disease leads to death, and how many people get sick [12]. In Milwaukee, many health disparities exist and vary across socio-economic and racial categories as it is one of the most segregated cities for African Americans and Whites by neighborhood [13]. There are many upstream factors in health disparities, one of which is differential access to and understanding of health information [14].

### Previous Works

With this context in mind, Science Cafés were intentionally designed to reach a broad spectrum of Milwaukee residents and to measure the impact of attending a Science Café on participants’ confidence in health and scientific literacy. Prior to this evaluation instrument there were no published instruments on measuring perceived changes in health and scientific literacy (PCHSL) in the general public. Data gathered from the first Science Café health series in 2013 showed that (1) attending a Science Café significantly improved attendees’ self-confidence levels in health and scientific literacy concepts; (2) repeat attendees did not have differences in their health and science self-confidence levels when compared with first time attendees; and (3) qualitative analysis of written comments supported the quantitative findings that attendees had a positive experience at the Science Café and suggested tools that would help them understand the information better, such as brief handouts with relevant Web sites [8].

Science Cafés were held as 2 distinct series throughout the year by the CTSI’s Community Engagement program over 4 years—Health Science Cafés (HSC) and Genomics Science Cafés (GSC). The focus of each series was slightly different; HSC series offered more emphasis on health-oriented topics, whereas GSC series focused more on science-oriented topics within a health framework. Differences in the topic and organization between series HSC and GSC created interest to determine if there was a variation in the PCHSL ratings by the attendees of each series.

### Current Study

For this study, data collection was completed using the same instrument from previous years. This afforded an opportunity to compare PCHSL between (1) series (HSC vs. GSC) and (2) to develop a model to show which factors (socioeconomic status (SES), education level) best predicted these changes.

For this study, we hypothesized that:

1. There would be a significant difference between the Series HSC and Series GSC participants’ PCHSL because of the multiple differences in teaching methods applied.
2. There would be a significant difference in the PCHSL by socio-economic status, as different levels of access to information exist across socio-economic statuses [12, 15, 16].
3. There would be a significant difference in the PCHSL by education level, as higher education influences overall literacy [15].

We anticipated results from this analysis would provide a data-driven framework for understanding both the strengths and gaps in the Science Café program in terms of reaching the communities we are attempting to impact.

## Methods

Predictors were collected from post-Science Café evaluations and included gender (male/female), age range (0–19 years, 20–39 years, 40–59 years, and 60+ years), highest education level achieved (recoded as: high school graduate/GED or less, some college or technical school, associate’s degree, bachelor degree, and graduate degree), SES group (defined by zip code conversion), and the Science Café series attended (HSC vs. GSC).

### Variable Descriptions

A detailed explanation and rationale of the method comparing PCHSL is offered in Ahmed *et al*.^2^ [8]. In evaluation methods design, the insights from Klatt and Taylor-Powell [17] were applied to use retrospective preratings compared with postintervention ratings in a design referred to as “post-then-pre.” The post-then-pre design is understood to capture learners understanding of where they actually were at pretest, on the basis of knowledge accumulated from the intervention, thus being in a better position to reflect on what they did not know at the pretest time point. Our previous study demonstrated that preratings compared with postratings yielded equivalent results to retrospective preratings compared with postratings for these data [8]. For clarity, we refer to the “then pre” measure as “retrospective pre.”

For the regression study, to better understand the sample and influence of SES status on outcomes, zip codes were recoded into high SES, medium SES, and low SES groups using the Milwaukee Health Report [13]. In this report, SES is determined on the basis of income and education levels following a previous approach used by Vila *et al*. [18].

### Study Sample

Data were collected from 23 Science Café event evaluations from 2013 to 2015 HSC and GSC series. All demographics from the sample population (n=457) are listed in Table 1.

**Table 1:** Study sample demographics comparison between series Health Science Cafés (HSC) and series Genomics Science Cafés (GSC)

Variables	Total n=457 (%)	Series HSC n=248 (%)	Series GSC n=209 (%)	*p* Value
Age category				0.729*
19 or below 39 years	91 (20.2)	43 (17.5)	48 (23.1)	
40–59 years	145 (32)	93 (37.9)	52 (25.1)	
60 years or older	217 (47.9)	109 (44.5)	108 (51.9)	
Missing	4	3	1	
Education				0.033*
High school graduate (GED or less)	18 (4.0)	10 (4.1)	8 (3.8)	
Some college or technical school	67 (14.7)	30 (12.2)	37 (17.7)	
Associate’s degree	25 (5.5)	16 (6.5)	9 (4.3)	
Bachelor degree	187 (41.1)	92 (37.4)	95 (45.5)	
Graduate degree	158 (34.7)	98 (39.8)	60 (28.7)	
Missing	2	2	0	
SES group				<0.001†
Low	53 (20.7)	43 (26.5)	10 (10.6)	
Medium	43 (16.8)	35 (21.6)	8 (8.5)	
High	160 (62.5)	84 (51.9)	76 (80.9)	
Missing	201	86	115	
Sex				0.006†
Male	144 (32.7)	63 (26.9)	81 (39.3)	
Female	296 (67.3)	171 (73.1)	125 (60.7)	
Missing	17	14	3	
First time attendee				0.010†
No	313 (71.5)	158 (66.4)	155 (77.5)	
Yes	125 (28.5)	80 (33.6)	45 (22.5)	
Missing	19	10	9	
Overall change‡				0.001§
Mean Δ±SD	0.7±0.8	0.6±0.8	0.9±0.8	
Missing	49	27	22	
1. General understanding				<0.001§
Mean postscore±SD‖	5.79±1.10	5.93±1.07	5.62±1.12	
Missing	42	25	17	
Mean Δ±SD	0.7±0.9	0.5±1.0	0.9±0.9	
Missing	51	28	23	
2. Ability to talk about topic				<0.001§
Mean postscore±SD	6±1	6±1	5±1	
Missing	45	28	17	
Mean Δ±SD	0.9±1.1	0.7±1.0	1.1±1.1	
Missing	53	30	23	
3. Ability to trust information				0.044§
Mean postscore±SD	5.67±1.25	5.95±1.08	5.34±1.36	
Missing	48	29	19	
Mean Δ±SD	0.8±1.0	0.7±1.0	0.9±1.0	
Missing	56	31	25	
4. Ability to find sources				0.073§
Mean postscore±SD	6±1	6±1	6±1	
Missing	45	26	19	
Mean Δ±SD	0.6±0.9	0.5±0.9	0.7±0.9	
Missing	55	29	26	
5. Ability to speak to researcher				0.050§
Mean postscore±SD	6±1	6±1	5±1	
Missing	46	25	21	
Mean Δ±SD	0.7±1.0	0.6±1.0	0.8±0.9	
Missing	54	27	27	

* Wilcoxon rank-sum test.

† χ^2^ Test.

‡ Overall change is the average of all 5 items.

§ *t* Test.

‖ Mean postratings are included for comparison between items in the health and science literacy scale and series, with these ratings one can calculate the retrospective preratings.

### Procedures

Research activities were approved by the Medical College of Wisconsin Institutional Review Board. Previous Café attendees were notified via email and flyers were distributed to advertise upcoming Café. After the Café, all participants were asked to complete an anonymous evaluation. The Café involved a medical professional or researcher engaging with the public in an informal, nonacademic environment (eg, Milwaukee Public Library located downtown). The speaker provided 20-minute background on the evening’s topic, notably *without* PowerPoint (in HSC series), followed by 30–60 minutes of open discussion among the audience and speaker. Series GSC often had PowerPoint and involved physical 3-dimensional models for participants to view and manipulate.

### Quantitative Analysis of Predictors of Change in Attendees’ PCHSL

Paired *t* tests were conducted to test for differences in retrospective preratings to postratings on the 5 areas of health and scientific literacy (full description in Ahmed *et al*. [8]) [17]. Next, demographic characteristics of Science Café attendees were compared between the 2 series using χ^2^ tests for nominal and Wilcoxon rank-sum test for ordinal outcomes. Finally, logistic regression analysis was conducted to identify independent predictors of change in attendees’ perceived confidence. The demographic variables measured in the evaluation were included in the final model even though some variables were not significant contributors (Tables 2 and 3). Potential interactions were tested among all predictors identified for inclusion into the model. Analyses were carried out using SAS 9.3 statistical software (SAS Institute Inc., Cary, NC, USA).

**Table 2:** Regression model with SES†

Parameter	Estimate	Standard error	*Pr>|t|*
Age category			0.999
60 and older vs. 40 vs. less than 60	0.007	0.144	0.959
Less than 40 vs. 40 vs. less than 60	0.001	0.161	0.993
Schooling completed			0.006**
Associate’s degree vs. high school graduate (GED or less)	−0.388	0.369	0.295
Bachelor degree vs. high school graduate (GED or less)	−0.850	0.321	0.009**
Graduate degree vs. high school graduate (GED or less)	−1.042	0.328	0.002***
Some college or technical school vs. high school graduate (GED or less)	−0.831	0.346	0.018*
Sex			0.834
Female vs. male	−0.026	0.126	
Series			0.207
GSC vs. HSC	0.163	0.129	
First time			0.143
Yes vs. no	0.193	0.131	
SES			0.007**
High vs. low	0.473	0.165	0.005***
Medium vs. low	0.557	0.195	0.005***

† SES and education are weakly correlated (Spearman ρ=0.25, *p*<0.001).

*p<0.05, **p<0.01, ***p<0.005.

**Table 3:** Regression model without SES

Parameter	Estimate	Standard error	*Pr>|t|*
Age category			0.967
60 and older vs. 40 vs. less than 60	−0.017	0.097	0.865
Less than 40 vs. 40 vs. less than 60	−0.030	0.124	0.807
Schooling completed			<0.0001*
Associate’s degree vs. high school graduate (GED or less)	−0.575	0.269	0.033***
Bachelor degree vs. high school graduate (GED or less)	−0.917	0.215	<0.0001*
Graduate degree vs. high school graduate (GED or less)	−1.056	0.216	<0.0001*
Some college or technical school vs. high school graduate (GED or less)	−0.990	0.224	<0.0001*
Sex			
Female vs. male	0.113	0.088	0.201
Series			
GSC vs. HSC	0.247	0.084	0.003**
First time			
Yes vs. No	0.026	0.098	0.791

*p<0.0001, **p<0.005, ***p<0.05.

## Results

### Does the Series of Science Café (HSC or GSC) Alter Participants’ PCHSL?

The populations in Series HSC versus Series GSC were significantly different in several demographic categories (see Table 1). The education profile was significantly different between series, with a higher education level in Series HSC compared with Series GSC. A significantly higher percentage of attendees were from low and medium SES groups in Series HSC than in Series GSC; Series GSC mainly had attendees from the high SES group. Finally, Series HSC had a significantly higher proportion of first time attendees than Series GSC.

Comparison of the postratings and retrospective preratings of PCHSL between Series HSC and Series GSC were significantly different, in Table 3 the mean postscore±SD is presented to show differences between series and individual items. When the health and scientific literacy scale was considered as individual items, 3 of the 5 were significantly different (items 1–3) with a larger mean difference observed in Series GSC than in Series HSC. Item 4 was not significantly different and item 5 was marginally significant between Series HSC and Series GSC.

### What Accounts for Participants’ PCHSL?

In light of SES groups being significantly different between Series HSC and GSC, we developed 2 possible regression models (SES Model and No SES Model) that accounted for participants’ PCHSL rating. As SES groups were only coded for those with zip codes in the City of Milwaukee, SES Model contains fewer participants (n=198), the No SES Model covers more data points (n=374) but has 1 less variable (SES).

In the SES Model (Table 2) significant predictors were level of education completed and SES group. Within the education level completed variable, there was a significant difference between (1) bachelor degree, (2) graduate degree, and (3) some college or technical school in comparison with high school graduate (GED or less). High school graduates showed about 1-point increase in PCHSL as compared with any of the 3 groups with higher levels of education. SES group was also a significant predictor: both high and medium SES groups showed about a 0.5-point increase in change in PCHSL rating compared with low SES group. SES and education level were weakly correlated (Spearman ρ=0.25, *p*<0.001). Overall, the SES Model accounted for 15% of the variability of the PCHSL. Age, sex, series, and first time attending were not significant predictors in this model.

The No SES Model (Table 3) looked at the following independent variables: age, schooling, sex, series, and first time attending. The significant predictors were education level completed and the series (HSC or GSC). Education levels completed and PCHSL ratings were negatively correlated suggesting that the more education level one had, the less likely they were to see an increase in their health and science literacy ratings. Within the education level completed variable, there was a significant difference between (1) bachelor degree, (2) graduate degree, (3) some college or technical school, and (4) associate’s degree in comparison with high school graduate (GED or less). There was a 1-point increase in change in PCHSL rating for high school graduates (GED or less) compared with the first 3 groups and 0.6-point increase in change in PCHSL compared with the 4th group, associate’s degree. Those participants attending Series GSC showed a 0.25-point increase in change in PCHSL levels as compared with the increase for those attending Series HSC. The No SES Model accounted for 10% of the variability of change in the PCHSL. Age, sex, and first time attending were not significant predictors in this model.

## Discussion

There are several factors affecting PCHSL. First, research shows individuals in low SES tend to seek less health information where individuals in high SES tend to seek more health information which suggests information seeking behavior and processing are closely tied with education and poverty levels [19, 20]. Levels of education in addition to educational attainment also factor into how literate an individual is in health and science and how they use the information [16, 19, 20]. In low health literacy individuals, there is difficulty in understanding and applying information provided to them by medical professionals thus making it more difficult to live a healthy lifestyle [21]. Building more opportunities for citizens and scientists to interact, as such through the Science Cafés can continue to gather what information is relevant to citizens and how they use the information in their daily lives to improve health [22].

Our hypotheses were confirmed by the findings in the results section:

1. In comparing the demographic variables and the PCHSL, it was confirmed that HSC and GSC series were significantly different.
2. In the SES Model, SES groups high and medium significantly contributed to increasing PCHSL.
3. In both models education level beyond high school significantly contributed to decreasing PCHSL.

## Limitations and Future Research

Although the study findings proved to be insightful for program development, there are several limitations. First, data on participants’ SES was limited to those living in the City of Milwaukee’s zip codes and was based on recoding data to 3 groups [13, 18]. Future evaluation forms have been modified to collect race and ethnicity data. In the future, we may consider developing our own SES index inclusive of more zip codes and gather income ranges from attendees at the Science Cafés. Our Community Engagement program has many community partners, future research would benefit from in-depth discussions with those partners about how to collect this type of information in a nonthreatening manner during Science Cafés and increase dialog on additional avenues for gathering sensitive information which may provide a more detailed view of different SES groups. Second, our target population still is the underserved; however, through the demographic analysis of both Series HSC and Series GSC we still have not fully reached the population of interest. In the next phase of the Science Cafés, we plan to develop a targeted communication plan to reach more individuals in the low and medium SES groups and across sociocultural lines. Our current strategy has been to deliver flyers to communities as well as sending email reminders. We have encountered numerous individuals who do not have email, still relying on postcard reminders or telephone calls. In addition to developing a more targeted communication plan, we also may need to change the topics of the Cafés, as leaders of community-based organizations we work with and not the individual community members themselves generated the current list of topics.

Series GSC does offer several insights for items to include in future programming to have higher impact in PCHSL. The educational tools used in Series GSC, such as 3-dimensional models, books, and repetition of material, are items that could be incorporated into Series HSC and future programming. Also, it may be beneficial for both series to have speakers present on a regular basis, so attendees can build rapport and the speaker has the opportunity to grow in their communication skills. Further additions to programming for both series would be to incorporate art or culturally, community-relevant forms of media, and plain language presentations, and written material for making science and health information more accessible. Series GSC briefly used a few drawings of cell structure to help explain information during the Cafés. Future Cafés could include an artist to illustrate discussions of what health issues are important to the community or have the speaker use the comic to help explain their research thus offering a new avenue of dissemination [21, 23].

## Conclusions

We have shown that the Science Café model impacts PCHSL over time. Future determinations of which factors in each series help to increase PCHSL and what factors of the population demographics influence the likelihood of change will lay a foundation for other researchers looking to impact underserved populations. We intend to work with our community partners to further expand our locations in low SES neighborhoods and present topics that interest all levels of SES, as our results suggest that those individuals from medium and high SES groups see an increase in PCHSL. In addition, education level beyond high school decreased PCHSL which suggests that it may be difficult to show an improvement in literacy after a certain level of educational attainment has been achieved. There is a notable dose effect with Series GSC and the use of multiple methods of teaching with focused discussion around the topic does increase PCHSL. Overall, dissemination of research findings to the public in a way that is beneficial to their health and well-being is of benefit to the entire community and researchers as well.

## Footnotes

1
As health literacy and science literacy have overlapping components, in this article the terms have been combined into a single conceptual entity here.

2
Full data set was analyzed with methods from Ahmed *et al.* and original finding that perceived changes in self-confidence in all 5 items on health and scientific literacy improve with this intervention, *p*<0.0001.

## References

[1] Beck F, et al Use of the internet as a health information resource among French young adults: results from a nationally representative survey. Journal of Medical Internet Research 2014; 16: e128. 24824164 10.2196/jmir.2934 PMC4051740

[2] Bennett IM, et al The contribution of health literacy to disparities in self-rated health status and preventive health behaviors in older adults. Annals of Family Medicine 2009; 7: 204–211. 19433837 10.1370/afm.940 PMC2682976

[3] Davis TC, Wolf MS. Health literacy: implications for family medicine. Family Medicine. 2004; 36: 595–598. 15343422

[4] Savolainen R. Everyday life information seeking: approaching information seeking in the context of ‘way of life’. Library & Information Science Research 1995; 17: 259–294.

[5] Yamashita T, Brown JS. Does cohort matter in the association between education, health literacy and health in the USA? *Health Promotion International* 2017; 32: 16–24. 10.1093/heapro/dat076 28180253

[6] Rooks RN, et al Health information seeking and use outside of the medical encounter: is it associated with race and ethnicity? Social Science & Medicine 2012; 74: 176–184. 22154611 10.1016/j.socscimed.2011.09.040

[7] Stilgoe J, Lock SJ, Wilsdon J. Why should we promote public engagement with science? Public Understanding of Science 2014; 23: 4–15. 24434705 10.1177/0963662513518154 PMC5753839

[8] Ahmed S, et al Science cafes: engaging scientists and community through health and science dialogue. Clinical and Translational Science 2014; 7: 196–200. 24716626 10.1111/cts.12153 PMC4410806

[9] Michener L, et al Aligning the goals of community-engaged research: why and how academic health centers can successfully engage with communities to improve health. Academic Medicine. 2012; 87: 285–291. 22373619 10.1097/ACM.0b013e3182441680 PMC3292771

[10] Peters HP. Gap between science and media revisited: scientists as public communicators. Proceedings of the National Academy of Sciences of the United States of America 2013; 110(Suppl. 3): 14102–14109. 23940312 10.1073/pnas.1212745110 PMC3752168

[11] Scheufele DA. Communicating science in social settings. Proceedings of the National Academy of Sciences of the United States of America 2013; 110(Suppl. 3): 14040–14047. 23940341 10.1073/pnas.1213275110 PMC3752169

[12] Dressler WW, Oths KS, Gravlee CC. Race and ethnicity in public health research: models to explain health disparities. Annual Review of Anthropology. 2005; 34: 231–252.

[13] Chen HYB, et al Milwaukee Health Report 2012: Health Disparaties in Milwaukee by Socioeconomic Status. Milwaukee, WI: Center for Urban Population Health, 2012.

[14] Brodie M, et al Health information, the Internet, and the digital divide. Health Affairs (Millwood) 2000; 19: 255–265. 10.1377/hlthaff.19.6.255 11192412

[15] Adler NE, Newman K. Socioeconomic disparities in health: pathways and policies. Health Affairs (Millwood) 2002; 21: 60–76. 10.1377/hlthaff.21.2.60 11900187

[16] Selwyn N. Apart from technology: understanding people’s non-use of information and communication technologies in everyday life. Technology in Society 2003; 25: 99–116.

[17] Klatt J, Taylor-Powell E. Program Development and Evaluation. Using the Retrospective Post- then-Pre Design, Quick Tips #27. University of Wisconsin-Extension, Madison, WI. Available at http://www.uwex.edu/ces/pdande/resources/index.html.

[18] Vila PM, et al Health disparities in Milwaukee by socioeconomic status. WMJ: Official Publication of the State Medical Society of Wisconsin 2007; 106: 366–372. 18030822

[19] James DC, et al Health literacy issues surrounding weight management among African American women: a mixed methods study. Journal of Human Nutrition and Dietetics 2015; 28(Suppl. 2): 41–49. 24890122 10.1111/jhn.12239

[20] Baker DW, et al The relationship of patient reading ability to self-reported health and use of health services. American Journal of Public Health 1997; 87: 1027–1030. 9224190 10.2105/ajph.87.6.1027 PMC1380944

[21] Davis TC, et al Health literacy and cancer communication. CA: A Cancer Journal for Clinicians 2002; 52: 134–149. 12018928 10.3322/canjclin.52.3.134

[22] Riesch H, Potter C. Citizen science as seen by scientists: methodological, epistemological and ethical dimensions. Public Understanding of Science 2014; 23: 107–120. 23982281 10.1177/0963662513497324

[23] Tedeschi B, Empinado H. WATCH: this young doctor’s cartoons bring smiles to young patients. *STAT News* [Internet], 2016. (https://www.statnews.com/2015/12/30/pediatrician-cartoon-sketch/)

